# How are US institutions implementing the new key information requirement?

**DOI:** 10.1017/cts.2020.1

**Published:** 2020-01-10

**Authors:** Jessica Mozersky, Matthew P. Wroblewski, Erin D. Solomon, James M. DuBois

**Affiliations:** Bioethics Research Center, Washington University School of Medicine, Box 8005, St. Louis, MO, USA

**Keywords:** Revised Common Rule, key information, review, implementation, regulatory guidance, informed consent, research ethics

## Abstract

Recent revisions to the Federal Policy for the Protections of Human Subjects require that informed consent documents begin with a “concise and focused presentation” of the key information a participant requires. Key information “must be organized and presented in a way that facilitates comprehension.” The regulations do not specify what information be included, nor how it must be presented to facilitate comprehension. It is unknown how institutions and Institutional Review Boards (IRBs) are interpreting the current regulations. We conducted a review of randomly sampled available key information templates at 46 US medical institutions to determine how they are implementing the new regulations.

## Introduction

On January 21, 2019, the long-awaited revisions to the Federal Policy for the Protections of Human Subjects (45 CFR 46), or Common Rule, came into effect. First proposed in 2011, and followed by two rounds of public comment to the Notice of Proposed Rulemaking (NPRM), the final revised Common Rule was published in January 2017.^1,2^ Actual implementation was delayed for another 2 years to provide institutions and researchers with adequate time to prepare for compliance with the updated regulations.

The revisions reflect changes in the kinds of research conducted and research subjects included in contemporary research.^1^ They include provisions to reduce the administrative burden for minimal risk studies, centralize Institutional Review Board (IRB) processes, and redefine exempt research and clinical trials. The revised Common Rule also includes new requirements for the informed consent process, which is the focus of this article.

A key aspect of the new guidance is that informed consent documents must now begin with a “concise and focused” presentation of the key information that is most likely to improve individual “understanding [of] the reasons why one might or might not want to participate in the research.”^3^ The addition of key information is meant to ensure the most important information that a “reasonable person” would want to know is contained upfront, rather than being buried within a document that contains “pages of tables” and “hundreds of risks.”^4^

Informed consent is a cornerstone of ethical research, yet evidence indicates that research participants frequently do not understand the information contained in consent documents ^4-6^ potentially compromising the fundamental right to autonomy informed consent is designed to uphold. Lack of comprehension can be a result of long overly complex documents containing technical jargon, and the fact that they frequently serve as legal documents to protect institutions, sponsors, and investigators from liability.^4,7^ Paasche-Orlow and colleagues found that consent templates provided by major medical schools fail to meet their own institutional standards for readability and had an average reading level 2–4 grades higher than the general US population.^5^ Therefore, it is perhaps unsurprising that the informed consent revisions “received substantial public support.”^4^

Notably, the Common Rule does not “strictly specify the types of information” that should be included in key information.^1^ At present, there are at least three guidance frameworks that a reasonable IRB might use (See Table 1). First, the preamble to the NPRM for the revised Common Rule contains five clusters of information that should be included in key information (hereafter called NPRM Preamble 5).^1^ Second, the final Common Rule contains a list of nine basic elements of informed consent although they refer to informed consent documents generally rather than key information specifically (hereafter called CR 9).^3^ Finally, Secretary’s Advisory Committee on Human Research Protections (SACHRP) has provided advice including nine questions to consider including in key information (hereafter called SACHRP 9), but official SACHRP guidance is forthcoming.^8^

**Table 1.** Regulatory guidance framework definitions

Notice of Proposed Rulemaking (NPRM) Preamble 5	The fact that consent is being sought for research and that participation is voluntary; The purposes of the research, the expected duration of the prospective subject’s participation, and the procedures to be followed in the research; The reasonably foreseeable risks or discomforts to the prospective subject; The benefits to the prospective subject or to others that may reasonably be expected from the research; and Appropriate alternative procedures or courses of treatment, if any, that might be advantageous to the prospective subject.
Common Rule (CR) 9	A statement that the study involves research, an explanation of the purposes of the research and the expected duration of the subject’s participation, a description of the procedures to be followed, and identification of any procedures that are experimental; A description of any reasonably foreseeable risks or discomforts to the subject; A description of any benefits to the subject or to others that may reasonably be expected from the research; A disclosure of appropriate alternative procedures or courses of treatment, if any, that might be advantageous to the subject; A statement describing the extent, if any, to which confidentiality of records identifying the subject will be maintained; For research involving more than minimal risk, an explanation as to whether any compensation and an explanation as to whether any medical treatments are available if injury occurs and, if so, what they consist of, or where further information may be obtained; An explanation of whom to contact for answers to pertinent questions about the research and research subjects’ rights, and whom to contact in the event of a research-related injury to the subject; A statement that participation is voluntary, refusal to participate will involve no penalty or loss of benefits to which the subject is otherwise entitled, and the subject may discontinue participation at any time without penalty or loss of benefits to which the subject is otherwise entitled; and One of the following statements about any research that involves the collection of identifiable private information or identifiable biospecimens: A statement that identifiers might be removed from the identifiable private information or identifiable biospecimens and that, after such removal, the information or biospecimens could be used for future research studies or distributed to another investigator for future research studies without additional informed consent from the subject or the legally authorized representative, if this might be a possibility; or A statement that the subject’s information or biospecimens collected as part of the research, even if identifiers are removed, will not be used or distributed for future research studies.
Secretary’s Advisory Committee on Human Research Protections (SACHRP) 9	What are the main reasons a subject will want to join this study? What are the main reasons a subject will not want to join this study? What is the research question the study is trying to answer? Why is it relevant to the subject? What aspects of research participation or this particular study are likely to be unfamiliar to a prospective subject, diverge from a subject’s expectations, or require special attention? What information about the subject is being collected as part of this research? What are the types of activities that subjects will do in the research? What impact will participating in this research have on the subject outside of the research? For example, will it reduce options for standard treatments? How will the subjects’ experience in this study differ from treatment outside of the study? In what ways is this research novel?

Furthermore, the Common Rule advises to organize information “in a way that facilitates comprehension” and be “no more than a few pages.”^1^ According to SACHRP, the “best solutions” for organizing and presenting key information that is easy to comprehend are not immediately apparent, but they note that there are many tools and guidelines that can help. Although official SACHRP guidance is lacking, evidence-based health communication best practices are well established and have demonstrated improved comprehension of informed consent documents.^6,9,10^ For instance, the use of plain language, formatting to include bullets and white space, increasing font size, and using visual aids have all been shown to increase understanding of complex materials.^10,11^

It is unknown how IRBs are interpreting the new key information regulations. We conducted a review of randomly sampled available key information templates and accompanying guidance at US medical institutions. Our review had three main goals: i) determine which guidance framework was used, if any; ii) describe the specific content; and iii) determine if any health communication best practices were included.

## Methods

The institutions were randomly selected from Clinical and Translational Science Awardees and Doctor of Medicine (MD)-granting medical schools in the USA (N = 150).^1^ A randomly selected sample of 47 of 150 key information documents generates an acceptable confidence interval and margin of error (CI = 0.9, ME = 0.1). We oversampled by randomly selecting 60 institutions as we anticipated that some institutions may not have publicly accessible guidance or may not have yet produced guidance given the newness of the regulations. Between February and May 2019, we extensively searched each institution’s IRB website for publicly available biomedical adult consent templates that included key information as well as any additional guidance provided pertaining to key information.

Documents were uploaded into Dedoose, a qualitative data coding and analysis software.

We used descriptive coding to categorize the content of documents.^11^ First, we determined if IRBs relied on existing guidance (NPRM Preamble 5^1^, SACHRP 9^8^, or CR 9^3^) as described above. Key information often contained regulatory guidance verbatim or nearly verbatim enabling us to readily determine what guidance was being followed. We counted the number of unique guidance items present in a given document for each framework and categorized it within a particular framework if the key information contained greater than 75% of the total items within a particular framework (i.e., 4 or more of the Preamble 5 topics, 7 or more of SACHRP 9 questions).

Second, we wanted to determine what specific content was contained within key information regardless of which regulatory framework was applied, especially as there is overlap between the frameworks (for instance, they all include an explanation of research). We coded the entire key information to identify content topics they contained, such as the purpose of research, risks, benefits, and voluntariness, that are associated with more than one regulatory framework. We also created a code to capture if key information could be waived under any circumstances.

Third, we wanted to explore whether key information documents included guidance on formatting, plain language, and other evidence-based communication best practices meant to enhance comprehension and readability.

We created a codebook with a priori codes but allowed for inductive coding to capture any new items that we did not anticipate. A priori regulatory codes were derived directly from regulatory guidance^1,3,8^, specific content codes were based on regulatory language and research ethics literature,^2,12,13^ and health communication best practice codes were derived from the literature.^6,9,10^ Our codebook including all operationalized codes is available in the supplementary materials.

In the first stage of coding, the entire team (JM, EDS, MPW, JMD) coded 10 of the key information documents as a group to ensure agreement on code application and definitions. One coder (MPW) then coded the remaining key information guidance documents bringing any coding queries to the group for discussion and resolution. Coding was conducted in Dedoose 8.2.14. Data were exported to Excel for analysis.

## Results

Of the 60 institutional websites sampled, 14 (23%) did not have key information or guidance available (7 were not accessible and 7 had accessible informed consent templates that did not include key information) yielding a final sample size of 46.

### Regulatory Guidance Contained in Key Information Templates

The majority of institutions relied on the NPRM Preamble 5 (Table 2). No institutions used SACHRP guidance on its own, and 2 used CR 9 elements. We identified two additional categories for the remaining documents: Hybrid/Other and Narrative Example. Hybrid/Other was the second most common type of guidance provided. Documents in this category did not adhere to one of the three regulatory frameworks but instead relied on components or combinations of the frameworks (for instance, combining Preamble 5 with SACHRP 9, or containing 3/5 of Preamble 5). Documents categorized in “Narrative Example” used a textual example of key information based on a hypothetical study rather than containing verbatim regulatory guidance.

**Table 2.** Regulatory guidance contained in key information templates

Regulatory guidance (n = 46)	#	%
Notice of Proposed Rulemaking (NPRM) Preamble 5	28	60.87
Hybrid/Other^a^	12	26.09
Narrative Example	4	8.69
Common Rule (CR) 9	2	4.34
Secretary’s Advisory Committee on Human Research Protections (SACHRP) 9	0	0.00

a 2 Hybrid/Other contained both SACHRP 9 and NPRM Preamble 5

### Specific Content Topics Contained in Key Information Templates

We also coded for specific content areas contained within key information regardless of what framework was applied. We began with a list of 19 a priori codes, all of which were present in key information at varying frequencies (Table 3). Table 3 also identifies whether the topic is included within existing regulatory guidance. The vast majority of documents contained the following topics: purpose of research, risks, duration, procedure, benefits, alternatives, and voluntariness—topics that are all encompassed by the NPRM Preamble 5—but beyond this there was wide variation in what topics were included within each key information. Notably, many of the SACHRP suggestions were not present in most key information.

**Table 3.** Frequencies and percentages of specific content topics contained in key information templates

Guidance	Content topic	#	%
C/P/S	Purpose or research is involved/Research questions or why relevant	46	100
C/P	Risks	45	98
C/P	Duration and time	44	96
C/P	Procedures	44	96
C/P	Benefits or lack thereof	41	89
C/P	Alternatives	34	74
C/P	Participation is voluntary	31	67
S	Main reasons NOT to join the study	16	35
S	Main reasons to join the study	14	30
C	Who to contact for questions or in case of injury	9	20
C	Confidentiality	7	15
I	Payment for participation	5	11
S	Types of activities that the subject will do	3	7
C	Compensation or medical expenses	2	4
S	How subjects’ experiences will differ from outside treatment	2	4
S	Impact on subject outside of the research	2	4
C	Information on biospecimen use for future research	2	4
S	Novel elements	2	4
S	Unfamiliar to prospective subject, unexpected, require special attention	1	2
S	What information is being collected from the subject	1	2

*Note.* C *=* Common Rule 9. I = Inductive. P = Preamble 5. S = SACHRP. Frequencies and percentages were calculated based on the number of institutions that addressed the key information element in their key information guidance (*n* = 46).

We also identified additional content areas inductively that were not on our a priori list. Only one inductive content area—payment for participation—was present in more than 10% of key information (Table 3). We identified 16 additional inductive content codes present in 12 institutions’ key information, but they are not reported here due to the infrequency they were found (<10%). The topics included conflicts of interest, prisoner research, phase I trials, termination circumstances, or unforeseeable risks.

Only eight documents (17%) contained conditions for waiving key information, and this generally involved waiving key information when a consent form was less than three to six pages in length.

### Guidance on Health Communication Best Practices

Over half of documents did not contain any guidance on health communication best practices (58.7%) (Table 4). The most common guidance provided was to use plain language (41%) followed by reading level (28%) and keeping to a certain page limit or word count (26%). Plain language guidance generally advised the use of simple or lay language, and avoiding technical jargon or defining it. When reading level was addressed, 10 out of 13 documents recommended 8th grade, 2 recommended 7th grade, and 1 recommended 6th grade reading level. When page length or word count guidance was provided (26%), suggestions included a single short paragraph, 2–3 paragraphs, no more than half or one page, 2–3 pages, a few pages, and in one case advised key information be no more than 1/3 the length of the entire consent form.

**Table 4.** Frequencies and percentages of health communication best practices guidance

Health communication guidance	#	%
No guidance	27	59
Plain language	19	41
Reading level	13	28
Page length or word count	12	26
Font size	8	17
Bullet points	4	9
Margin or white space	3	7
Graphics or figures	2	4
Table	2	4

*Note.* Frequencies and percentages were calculated based on the number of institutions that addressed the key information element in their key information guidance (*n* = 46).

## Discussion

The majority of key information sampled rely on the NPRM Preamble 5, or a variation that included elements primarily from the Preamble 5. Very few documents used CR 9, and no documents relied on SACHRP 9 alone. The individual content topics contained within documents, regardless of which regulatory guidance was being used, were also primarily derived from the Preamble 5.

Notably, SACHRP states the following about the Preamble 5 “Although from a compliance perspective, the fact that these elements of consent are listed in the preamble makes them attractive as a safe harbor of sorts, SACHRP believes such a use may not be in keeping with the intent of the regulatory change.”^8^ Key information templates do not currently reflect SACHRP’s suggestion to include “new information that is not a required element of consent…in order to best facilitate informed decision making.”^8^

Over half of documents did not contain any guidance on health communication best practices.^6,9,10^ Beyond plain language, the majority of documents did not address communication best practices. Over 70% of the documents did not address reading level. Given that 43% of adults living in the USA have basic (simple text reading ability) or below basic (nonliterate to very simple text reading) literacy skills,^14^ this suggests consent forms will continue to be written at far higher grade levels than the average US population.^5^ Furthermore, those IRBs that addressed length tended to require key information sections that would be so brief as to preclude the use of communication best practices, which require the use of larger fonts, bullets, increased white space, and tables or figures.

Our findings suggest that key information documents are relying on the minimum standards laid out in the federal regulations for what information to include rather than the more radical changes suggested by SACHRP to include new information that will “fundamentally change and improve the consent process.”^8^ In fact, no institutions relied solely on the SACHRP 9 questions suggested for inclusion in key information. In addition, most institutions offer no guidance on plain language, reading level, or the formatting of information (such as font and margin size). Institutions could improve comprehension of key information by providing investigators with evidence-based guidance on how to maximize the readability of information.

### Limitations

The documents reported here were collected several months after the Revised Common Rule went into effect and therefore represent institutions’ first attempts at complying with the new key information requirement.

## Footnotes

1 The list was compiled using the Association of American Medical Colleges members directory of MD granting medical schools (https://members.aamc.org/eweb/DynamicPage.aspx?site=AAMC&webcode=AAMCOrgSearchResult&orgtype=Medical%20School) and all institutions with Clinical and Translational Science Awards (CTSA) (https://ncats.nih.gov/ctsa/about/hubs).Three recently awarded CTSAs and three MD granting medical schools were not included in our list.

## Supplementary material

For supplementary material accompanying this paper visit https://doi.org/10.1017/cts.2020.1. click here to view supplementary material
