# Supplementary material for: How are US institutions implementing the new key information requirement?
Source: J Clin Transl Sci. 2020 Jan 10;4(4):365–9. doi: 10.1017/cts.2020.1 (PMC7681124; doi:10.1017/cts.2020.1)
Supplement: Supplementary file 1 [file S2059866120000011sup001.docx]

**Supplemental Materials**

**Key Information Codebook**

| **Code** | **Definition** |
| --- | --- |
| **Regulatory Guidance Frameworks** | |
| NPRM Preamble 5 | Code if document contains 4 or more of the following Preamble 5 elements:   1. the fact that consent is being sought for research and that participation is voluntary; 2. the purposes of the research, the expected duration of the prospective subject’s participation, and the procedures to be followed in the research; 3. the reasonably foreseeable risks or discomforts to the prospective subject; 4. the benefits to the prospective subject or to others that may reasonably be expected from the research; and 5. appropriate alternative procedures or courses of treatment, if any, that might be advantageous to the prospective subject. |
| CR 9 | Code if document contains 7 or more of the following CR 9 elements:   1. A statement that the study involves research, an explanation of the purposes of the research and the expected duration of the subject's participation, a description of the procedures to be followed, and identification of any procedures that are experimental; 2. A description of any reasonably foreseeable risks or discomforts to the subject; 3. A description of any benefits to the subject or to others that may reasonably be expected from the research; 4. A disclosure of appropriate alternative procedures or courses of treatment, if any, that might be advantageous to the subject; 5. A statement describing the extent, if any, to which confidentiality of records identifying the subject will be maintained; 6. For research involving more than minimal risk, an explanation as to whether any compensation and an explanation as to whether any medical treatments are available if injury occurs and, if so, what they consist of, or where further information may be obtained; 7. An explanation of whom to contact for answers to pertinent questions about the research and research subjects' rights, and whom to contact in the event of a research-related injury to the subject; 8. A statement that participation is voluntary, refusal to participate will involve no penalty or loss of benefits to which the subject is otherwise entitled, and the subject may discontinue participation at any time without penalty or loss of benefits to which the subject is otherwise entitled; and 9. One of the following statements about any research that involves the collection of identifiable private information or identifiable biospecimens: 10. A statement that identifiers might be removed from the identifiable private information or identifiable biospecimens and that, after such removal, the information or biospecimens could be used for future research studies or distributed to another investigator for future research studies without additional informed consent from the subject or the legally authorized representative, if this might be a possibility; or 11. A statement that the subject's information or biospecimens collected as part of the research, even if identifiers are removed, will not be used or distributed for future research studies. |
| SACHRP 9 | Code if document contains 7 or more of the following SACHRP 9 questions:   1. What are the main reasons a subject will want to join this study? 2. What are the main reasons a subject will not want to join this study? 3. What is the research question the study is trying to answer?  Why is it relevant to the subject? 4. What aspects of research participation or this particular study are likely to be unfamiliar to a prospective subject, diverge from a subject’s expectations, or require special attention? 5. What information about the subject is being collected as part of this research? 6. What are the types of activities that subjects will do in the research? 7. What impact will participating in this research have on the subject outside of the research? For example, will it reduce options for standard treatments? 8. How will the subjects’ experience in this study differ from treatment outside of the study? 9. In what ways is this research novel? |
| Hybrid/Other | Documents are coded here in the following cases:   1. Two regulatory frameworks are present, i.e., greater than 75% of the total items from a framework for two frameworks; OR 2. Does not fall into any of the other frameworks |
| Narrative example | Documents are coded here if the key information sheet does not provide any of the 3 regulatory guidance above but instead includes a textual example of key information based on a hypothetical study |
| **A Priori Content Topics** | |
| Purpose or research is involved/Research questions or why relevant | Guidance advises including information on the purpose of the study, the fact that research is involved, the study’s research questions, or why the study is relevant for the participant |
| Risks | Guidance advises including information on risks |
| Duration and time | Guidance advises including information on duration and time |
| Procedures | Guidance advises including information on procedures |
| Benefits or lack thereof | Guidance advises including information on benefits or lack thereof |
| Alternatives | Guidance advises including information on alternatives |
| Participation is voluntary | Guidance advises including information on the fact that participation is voluntary |
| Main reasons not to join the study | Guidance advises including information on the main reasons not to join the study |
| Main reasons to join the study | Guidance advises including information on the main reasons to join the study |
| Who to contact for questions or in case of injury | Guidance advises including information on who to contact for questions or in case of injury |
| Confidentiality | Guidance advises including information on confidentiality |
| Types of activities the subject will do | Guidance advises including information on types of activities that the subject will do |
| Compensation or medical expenses | Guidance advises including information on compensation or medical expenses that might be incurred |
| How subjects’ experiences will differ from outside treatment | Guidance advises including information on how subjects’ experience will differ from outside treatment |
| Impact on subject outside of the research | Guidance advises including information on the impact on the subject outside of the research |
| Information on biospecimen use for future research | Guidance advises including information on the collection of identifiable private information or identifiable biospecimens |
| Novel elements | Guidance advises including information on novel elements of the research |
| Unfamiliar to prospective subject, unexpected, require special attention | Guidance advises including information on aspects of research participation or this particular study that are unfamiliar to a prospective subject, unexpected, or require special attention |
| What information is being collected from the subject | Guidance advises including information on what information is being collected from the subject |
| Condition for Applicability | Guidance specifies the conditions when key information sheets are required |
| **Inductive Content Topics** | |
| Other | Guidance advises any content topic not included in our a priori list |
| **Communication and Formatting** | |
| Plain language | Guidance suggests using plain language |
| Reading level | Guidance suggests using a certain reading level |
| Page length or word count | Guidance suggests using a certain page length or word count |
| Font size | Guidance suggests using a certain font size |
| Bullet points | Guidance suggests using bullet points |
| Margin or white space | Guidance suggests increasing margins or white space |
| Graphics or figures | Guidance suggests using graphics or figures |
| Table | Guidance suggests using tables |
